# Immunoprofiling Reveals GATA3 as a Prognostic Marker in Transformed Mycosis Fungoides

**DOI:** 10.1111/1346-8138.70070

**Published:** 2025-12-18

**Authors:** Bruno de Castro e Souza, Denis Miyashiro, Jade Cury‐Martins, Neusa Yuriko Sakai Valente, Luiz Fernando Ferraz da Silva, José Antonio Sanches

**Affiliations:** ^1^ Department of Dermatology University of São Paulo Medical School São Paulo Brazil; ^2^ Department of Pathology University of São Paulo Medical School São Paulo Brazil

## Abstract

Transformed mycosis fungoides (TMF) is a rare, aggressive variant of cutaneous T‐cell lymphoma characterized by the presence of large neoplastic cells and poor clinical outcomes. A retrospective cohort of 22 TMF patients was analyzed using immunohistochemistry on formalin‐fixed, paraffin‐embedded (FFPE) tissue for GATA3 (*n* = 20), T‐bet (*n* = 22), and STAT3 (*n* = 22). Expression was quantified by image analysis integrated optical density per total area (IOD/area), standardized by *z*‐score and correlated with survival. Seventeen patients with complete data underwent unsupervised clustering (*k*‐means) and principal component analysis (PCA) based on marker expression profiles. High GATA3 expression was strongly associated with worse prognosis (median OS: 8.6 months vs. 41.7 months, *p* = 0.00094). T‐bet and STAT3 expressions showed no significant individual association with survival. Clustering analysis revealed three distinct immunoprofiles: (1) low expression of all markers (intermediate survival, 28.1 months), (2) high STAT3 and T‐bet expressions with intermediate GATA3 expression (longest survival, 53.1 months), and (3) high GATA3 expression with low STAT3 and T‐bet expressions (poorest survival, 9.5 months). GATA3 is a robust prognostic marker in TMF, identifying patients with particularly poor outcomes. Its elevated expression delineates a Th2‐skewed, immunosuppressive phenotype that may inhibit Th1/Th17 pathways via transcriptional repression. Integrative profiling reveals immunobiological subgroups with divergent prognoses, supporting GATA3 as a potential tool for risk stratification and a candidate for targeted intervention in TMF.

## 1 Introduction

Transformed mycosis fungoides (TMF) is a rare but aggressive variant of classic mycosis fungoides, defined histologically by the presence of large tumor cells comprising more than 25% of the infiltrate [1, 2, 3]. This transformation is frequently associated with CD30 expression, increased proliferative activity, and a rapidly progressive clinical course [1, 2, 3]. While the poor prognosis of TMF is well established, survival outcomes remain heterogeneous, suggesting that additional molecular markers may influence disease progression and patient outcomes.

Recent interest has turned to transcriptional regulators of T‐cell polarization as potential prognostic biomarkers. GATA3, T‐bet, and STAT3 are central to Th2, Th1, and Th17 cell lineage commitment, respectively, and have been implicated in the biology and immune environment of peripheral T‐cell lymphomas (PTCL) [4, 5]. This study aimed to evaluate the expression of these markers in TMF and their relationship to overall survival (OS) and immunobiological clustering.

## 2 Objectives

The objectives of this study were to quantify the expression of GATA3, T‐bet, and STAT3 in patients with TMF, to examine the association of these transcription factors with OS, and to identify distinct immunobiological subgroups through integrative analysis of their expression patterns.

## 3 Methods

### 3.1 Cohort Description

A total of 22 patients diagnosed with transformed mycosis fungoides to large‐cell lymphoma were retrospectively identified from a tertiary academic center between 2012 and 2023 (Table 1). All cases had histologically confirmed transformation in skin biopsies, with and available formalin‐fixed, paraffin‐embedded (FFPE) tissue samples.

**TABLE 1:** Baseline clinical characteristics of the 22 patients diagnosed with transformed mycosis fungoides (2012–2023). Values are expressed as median and interquartile range [IQR] for continuous variables, and absolute number (percentage) for categorical variables.

Variable	Value
Sex—male/female	9/13
Age at transformation—median [IQR], years	51.8 [40.5–66.8]
Follow‐up time—median [IQR], months	22.0 [7.6–40.0]
Deaths during follow‐up, *n* (%)	16 (72.7%)
Clinical stage at transformation	IIB: 15/IB: 4/IA: 1/IVA1: 1/IVA2: 1

### 3.2 Immunohistochemistry and Quantification

Immunohistochemical staining for GATA3 (clone L50‐823, mouse monoclonal antibody, *n* = 20; Roche), T‐bet (clone EPR9301, rabbit monoclonal antibody, *n* = 22; Abcam), and STAT3 (pSTAT3; Tyr705, clone EP2147Y, rabbit monoclonal antibody, *n* = 22; Abcam) was performed using standardized automated protocols. Digital images were acquired under uniform conditions and standardized for brightness, contrast, and color. Quantification was done using Image‐Pro Plus software (Media Cybernetics), and expression was calculated as integrated optical density per total area (IOD/area). As the software does not allow distinction between malignant and reactive lymphocytes at the single‐cell level, regions of interest were selected by the pathologist in areas with the highest density of transformed tumor cells, thereby minimizing potential bias from reactive components.

Expression values were standardized using *z*‐score transformation. For categorical analyses, patients were dichotomized based on median expression values.

### 3.3 Integrative Analysis

Seventeen patients with complete data for all three markers were included in the integrative clustering analysis. *Z*‐score‐standardized values of GATA3, T‐bet, and STAT3 were used to generate a heatmap. Unsupervised clustering was performed using the *k*‐means algorithm (*k* = 3), and principal component analysis (PCA) was applied for visualization. Point size in PCA plots was scaled to OS.

### 3.4 Statistical Analysis

OS was defined from the date of histologic transformation to death or last follow‐up. Kaplan–Meier survival curves were compared using the log‐rank test. Multivariable analysis of OS was performed using a Cox proportional hazards model including GATA3 expression as the main variable, adjusted for age at transformation, serum LDH level, and clinical stage. Hazard ratios (HRs) with 95% confidence intervals (CIs) were calculated. Analyses were conducted in R (v.4.2.0). Statistical significance was defined as *p* < 0.05.

## 4 Results

### 4.1 GATA3 Expression and Prognosis

**FIGURE 1:**
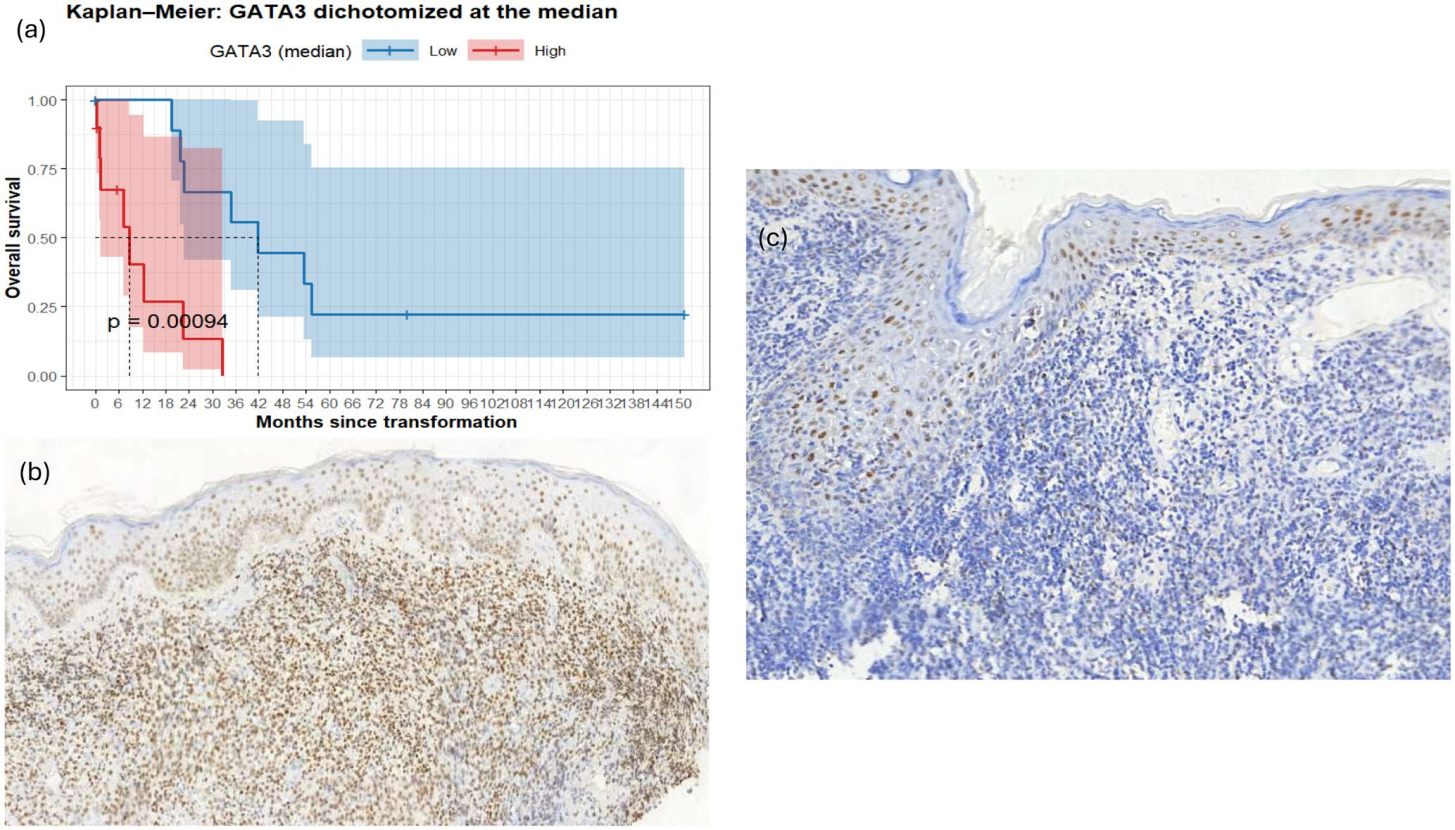
(a) Kaplan–Meier survival analysis of overall survival stratified by GATA3 expression dichotomized at the median. Patients with high GATA3 expression had significantly worse outcomes (median OS: 8.6 months vs. 41.7 months, *p* = 0.00094). (b) Representative immunohistochemistry of a case with high GATA3 expression, showing diffuse and intense nuclear staining of atypical lymphocytes. (c) Representative case with low GATA3 expression, with sparse and weak nuclear positivity restricted to scattered reactive lymphocytes and keratinocytes.

High GATA3 expression (*n* = 10) was significantly associated with worse prognosis: median OS was 8.6 months versus 41.7 months in the low‐expression group (*n* = 10; *p* = 0.00094). None of the high‐expression patients survived beyond 5 years, whereas the 5‐year survival rate in the low‐expression group was 22.2% (Figure 1a). Representative immunohistochemical staining for GATA3 in cases with high and low expressions is shown in Figure 1b,c, respectively. In multivariable analysis including age at transformation, LDH levels, and clinical stage, high GATA3 expression remained independently associated with worse prognosis (HR = 10.56, 95% CI 1.54–72.36, *p* = 0.016).

### 4.2 T‐Bet and STAT3 Expressions

T‐bet and STAT3 were not significantly associated with OS. For T‐bet, the median OS was 34.8 months in the low‐expression group and 22.7 months in the high‐expression group (*p* = 0.62). STAT3 expression yielded a median OS of 19.5 months (low) and 22.4 months (high), with no statistical significance (*p* = 0.24). However, 30% of patients with high STAT3 expression survived beyond 5 years, compared to none in the low‐expression group.

### 4.3 Immunobiological Clustering

**FIGURE 2:**
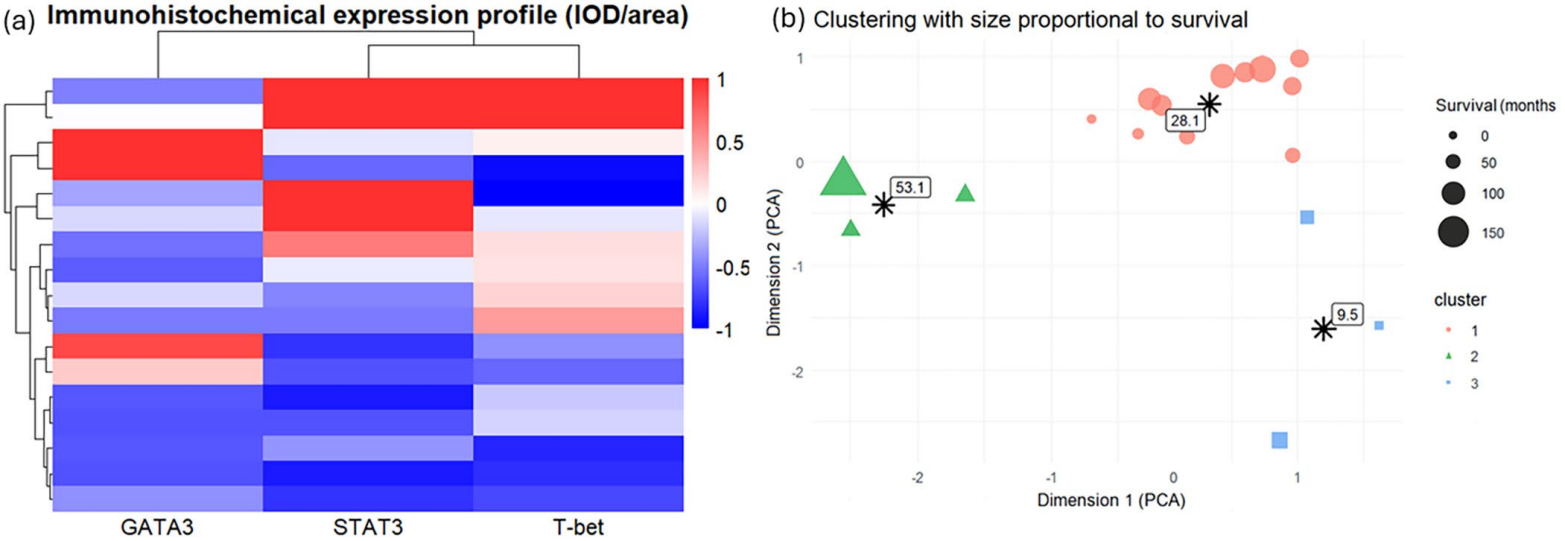
(a) Immunohistochemical expression profile of GATA3, STAT3, and T‐bet in patients with transformed mycosis fungoides. Heatmap based on IOD/area ratio for each marker, standardized by *z*‐score. Rows represent individual patients and columns represent markers. Color intensity ranges from blue (low relative expression) to red (high relative expression). The dendrogram indicates patient clustering based on expression similarity. Distinct subgroups with predominant GATA3 or T‐bet/STAT3 expression suggest different immunological polarizations. (b) Clustering of patients based on combined expressions of GATA3, T‐bet, and STAT3, visualized in two dimensions by PCA. Each point represents a patient, colored and shaped according to the *k*‐means cluster assignment (*k* = 3). Point size is proportional to overall survival (in months) since transformation. Black asterisks indicate cluster centroids, annotated with the corresponding mean survival. Cluster 1 (red) shows low expression of all three markers and intermediate survival. Cluster 2 (green) is characterized by high T‐bet and STAT3 expressions and longer survival. Cluster 3 (blue) includes patients with elevated GATA3 expression and the shortest survival. IOD, integrated optical density; PCA, principal component analysis.

To explore immunological expression profiles, a heatmap was constructed using z‐score–standardized IOD/area values for the 17 patients with complete data on GATA3, T‐bet, and STAT3 (Figure 2a). Each row represents a patient, and each column corresponds to a transcription factor. Color intensity ranges from blue (relatively low expression within that marker's distribution) to red (relatively high expression), enabling visual identification of contrasting expression patterns. The heatmap revealed distinct immunological profiles: one subgroup exhibited high GATA3 expression with low expression of T‐bet and STAT3, while another showed the opposite—coexpression of T‐bet and STAT3 with intermediate GATA3.

To better define these patterns, we conducted an unsupervised clustering analysis. Three distinct clusters emerged (Figure 2b):

- Cluster 1 (*n* = 11; mean OS: 28.1 months): characterized by globally lower relative expression of all markers compared with the other groups.
- Cluster 2 (*n* = 3; mean OS: 53.1 months): high STAT3 and T‐bet, but intermediate GATA3 expressions.
- Cluster 3 (*n* = 3; mean OS: 9.5 months): high GATA3, and low STAT3 and T‐bet expressions.

These immunobiological subgroups showed divergent survival outcomes, reinforcing the clinical relevance of the underlying immune expression patterns.

## 5 Discussion

The pathophysiology of MF involves progressive remodeling of the immune microenvironment, transitioning from a Th1‐dominant to a Th2‐dominant profile as the disease advances. This functional polarization directly impacts antitumor immunity and facilitates immune escape in advanced stages [6, 7].

In this context, GATA3 and T‐bet represent the master transcriptional regulators of Th2 and Th1 differentiation, respectively. GATA3 drives IL‐4, IL‐5, and IL‐13 expressions, contributing to allergic and immunosuppressive responses, whereas T‐bet induces IFN‐γ and cytotoxic signaling. The gradual increase in GATA3 and decline of T‐bet expression during MF progression has been identified as a key mechanism of immune escape [6, 7].

In our cohort of patients with transformed MF, immunohistochemical evaluation of GATA3, T‐bet, and STAT3 expressions allowed for the definition of distinct biological subgroups with differential survival outcomes. GATA3 expression alone showed a strong association with poor prognosis, with high‐expression patients having a median OS of 8.6 months versus 41.7 months in the low‐expression group (*p* = 0.00094). Importantly, in multivariable analysis adjusted for age, LDH elevation, and clinical stage, high GATA3 expression persisted as an independent prognostic factor (HR = 10.56, 95% CI 1.54–72.36, *p* = 0.016), underscoring the robustness of its association with adverse outcomes.

Survival outcomes in TMF have been reported with considerable variability across different cohorts, ranging from < 2 years to nearly 5 years of median OS, depending on study design, patient composition, and therapeutic strategies [8, 9]. Within this context, the survival observed in our series is consistent with the broad spectrum of outcomes previously described. What is particularly noteworthy in our cohort is the markedly inferior prognosis of patients with high GATA3 expression underscoring its role as a high‐risk biomarker. Differences in clinical stage and heterogeneity in treatment approaches may further contribute to the variability in survival across published studies.

These findings are consistent with previous studies in classic MF, where GATA3 is associated with tumor stage progression, higher genetic burden (e.g., TP53 mutations), and Th2‐dominant subtypes with low cytotoxic activity. Recent data also demonstrated that high GATA3 expression independently predicts worse progression‐free and OS even in early‐stage classical MF, reinforcing its prognostic significance across the disease spectrum [10]. Transcriptomic analyses also highlight the enrichment of tumor‐associated macrophages (M2 phenotype) in GATA3+ tumors, reinforcing the link to immune suppression [11, 12].

Despite its role in Th2 polarization, GATA3's impact appears to extend beyond Th1 antagonism, as T‐bet expression was not significantly associated with prognosis in our series. This is supported by studies in PTCL, where GATA3+ subgroups consistently demonstrate worse outcomes than TBX21+ counterparts, even in the context of high T‐bet expression [11, 12]. A similar immunophenotypic dichotomy was also reported in indolent adult T‐cell leukemia/lymphoma with cutaneous lesions, where the GATA3 subtype correlated with significantly shorter survival compared to the TBX21 subtype, further reinforcing the adverse prognostic role of GATA3 across distinct T‐cell lymphoma entities [13].

STAT3 is a transcription factor activated by IL‐6 and IL‐21, essential for Th17 differentiation through induction of RORγt (RORC) [14]. In our study, we specifically assessed the phosphorylated form of STAT3 (pSTAT3, Tyr705) and considered only nuclear staining as positive, since nuclear translocation reflects pathway activation. While STAT3 alone did not significantly affect survival (*p* = 0.24), its integration with GATA3 and T‐bet expressions revealed immunobiological clusters with distinct prognoses: Cluster 3, with high GATA3 and low STAT3/T‐bet expressions, had the worst outcome (9.5 months), suggesting a highly immunosuppressive environment.

The inverse relationship between GATA3 and STAT3 observed in our heatmap mirrors existing evidence of mutual transcriptional repression: GATA3 can bind the STAT3 promoter and inhibit its expression, while IL‐6‐driven STAT3 activation can suppress GATA3 [15, 16]. This interaction likely reduces RORγt activity, limiting Th17‐mediated inflammation.

This model aligns with the study by Marques‐Piubelli et al., which found that low RORγt expression in mycosis fungoides correlated with worse survival, an exhausted immune phenotype, and elevated PD‐1 expression [5].

This study has limitations that should be acknowledged. The relatively small sample size, although reflective of the rarity of TMF, limits statistical power and constrains the generalizability of our findings. In addition, external validation in independent cohorts was not performed, and future multicenter studies will be essential to confirm the prognostic impact of GATA3 expression and to strengthen the reproducibility of our observations.

In summary, GATA3 emerges as a multifaceted high‐risk marker in TMF, integrating three dimensions of tumor aggressiveness: (1) Th2 functional bias; (2) suppression of Th1/Th17 axes; and (3) association with genetic alterations and immunosuppressive remodeling of the tumor microenvironment. Altogether, these findings help elucidate the clinical heterogeneity of TMF and highlight GATA3 as a promising marker for risk stratification and a potential therapeutic target.

## Funding

This work was supported by Fundo de Apoio à Dermatologia de São Paulo (FUNADERSP).

## Ethics Statement

All necessary approvals were obtained from our ethics committee, and the study was undertaken in accordance with the Declaration of Helsinki.

## Consent

All patients provided written informed consent for personal data analysis for research purposes.

## Conflicts of Interest

The authors declare no conflicts of interest.

## Data Availability

The data that support the findings of this study are available on request from the corresponding author. The data are not publicly available due to privacy or ethical restrictions.
